# Association of obstructive sleep apnea syndrome with polycystic ovary syndrome through bidirectional Mendelian randomization

**DOI:** 10.3389/fmed.2024.1429783

**Published:** 2024-06-28

**Authors:** Peijun Liu, Qin Zhang, Haitao Ding, Hua Zou

**Affiliations:** ^1^Department of Respiratory and Critical Care Medicine, The Central Hospital of Enshi Tujia and Miao Autonomous Prefecture, Enshi, China; ^2^Department of Nursing, The Central Hospital of Enshi Tujia and Miao Autonomous Prefecture, Enshi, China; ^3^Department of Emergency, The Central Hospital of Enshi Tujia and Miao Autonomous Prefecture, Enshi, China

**Keywords:** PCOS, OSAS, Mendelian randomization, causal inference, GWAS

## Abstract

**Background:**

Observational studies have established a link between polycystic ovary syndrome (PCOS) and obstructive sleep apnea syndrome (OSAS), with obesity being a significant confounding factor that complicates the understanding of causality. This study seeks to clarify the causal relationship by utilizing bidirectional two-sample Mendelian randomization (MR) analysis.

**Methods:**

A bidirectional MR strategy was implemented to investigate the potential causal relationship between PCOS and OSAS. Instrumental variables (IVs) for PCOS were sourced from a dataset comprising 3,609 cases and 229,788 controls. For OSAS, statistical data were obtained from a genome-wide association study (GWAS) involving 38,998 subjects, alongside a control group of 336,659 individuals. Our MR analysis utilized several methods, including inverse variance weighted (IVW), weighted mode, weighted median, simple mode, and MR-Egger, primarily focusing on the IVW technique. Sensitivity tests were conducted to ensure the robustness of our findings.

**Results:**

Utilizing the IVW method, we identified a notable causal association from OSAS to PCOS, with an odds ratio (OR) of 1.463 and a 95% confidence interval (CI) of 1.086–1.971 (*p* = 0.012). In the opposite direction, PCOS also appeared to significantly affect OSAS development, indicated by an OR of 1.041 and a 95% CI of 1.012–1.072 (*p* = 0.006). The MR-Egger intercept test showed no evidence of directional pleiotropy, affirming the credibility of our causal findings (*p* > 0.05).

**Conclusion:**

This study suggests a bidirectional causal relationship between PCOS and an increased risk of OSAS. These insights could guide future screening and prevention strategies for both conditions.

## 1 Introduction

Polycystic ovary syndrome (PCOS) is a metabolic disorder affecting about 8–13% of women, marked by issues with ovulation, elevated androgen levels, and insulin resistance (1). This widespread endocrine disorder is marked by high androgen levels and menstrual disturbances, substantially increasing the risk of insulin resistance and metabolic syndrome. Factors like anovulation, hypothalamic dysfunctions, and menstrual irregularities can lead to diminished or absent progesterone in PCOS patients. The activity of estrogens, affected by their composition and metabolic traits in the body, is reduced in women with PCOS. Common manifestations of PCOS encompass hirsutism, obesity, acne, menstrual anomalies, and infertility (2, 3).

The worldwide occurrence of obstructive sleep apnea syndrome (OSAS) falls within a range of 9–38%, and it is progressively increasing due to the rising rates of obesity and the aging global population (4). Diagnosing OSAS involves identifying primary symptoms, including pronounced daytime fatigue, loud snoring, observed interruptions in breathing during sleep, and sleep analysis findings that reveal significant disruptions in breathing, specifically the apnea hypopnea index (AHI). In instances lacking overt symptoms, a diagnosis can still be established if the apnea-hypopnea index exceeds 15 episodes per hour (5, 6). Notably, the symptomatology in women with OSAS, characterized by difficulties in waking, insomnia, morning fatigue, headaches, depression, and anxiety, tends to be subtler and thus more frequently disregarded. This oversight contributes to a systemic underreporting of OSAS prevalence among females (7).

Mendelian randomization (MR) emerges as a crucial instrument in epidemiology, targeting the discovery of causal relationships between potential risk elements and health outcomes utilizing genetic variations as surrogates (8). Distinguished from traditional observational research, single nucleotide variations (SNPs) were identified via genome wide association studies (GWAS) functioning in the role of instrumental variables (IVs). This approach protects against confounding due to the random allocation of genetic variations at birth (9, 10). Our research endeavors to unravel the causative dynamics between PCOS and OSAS through bidirectional MR analysis.

## 2 Materials and methods

### 2.1 Study design

**Figure 1:**
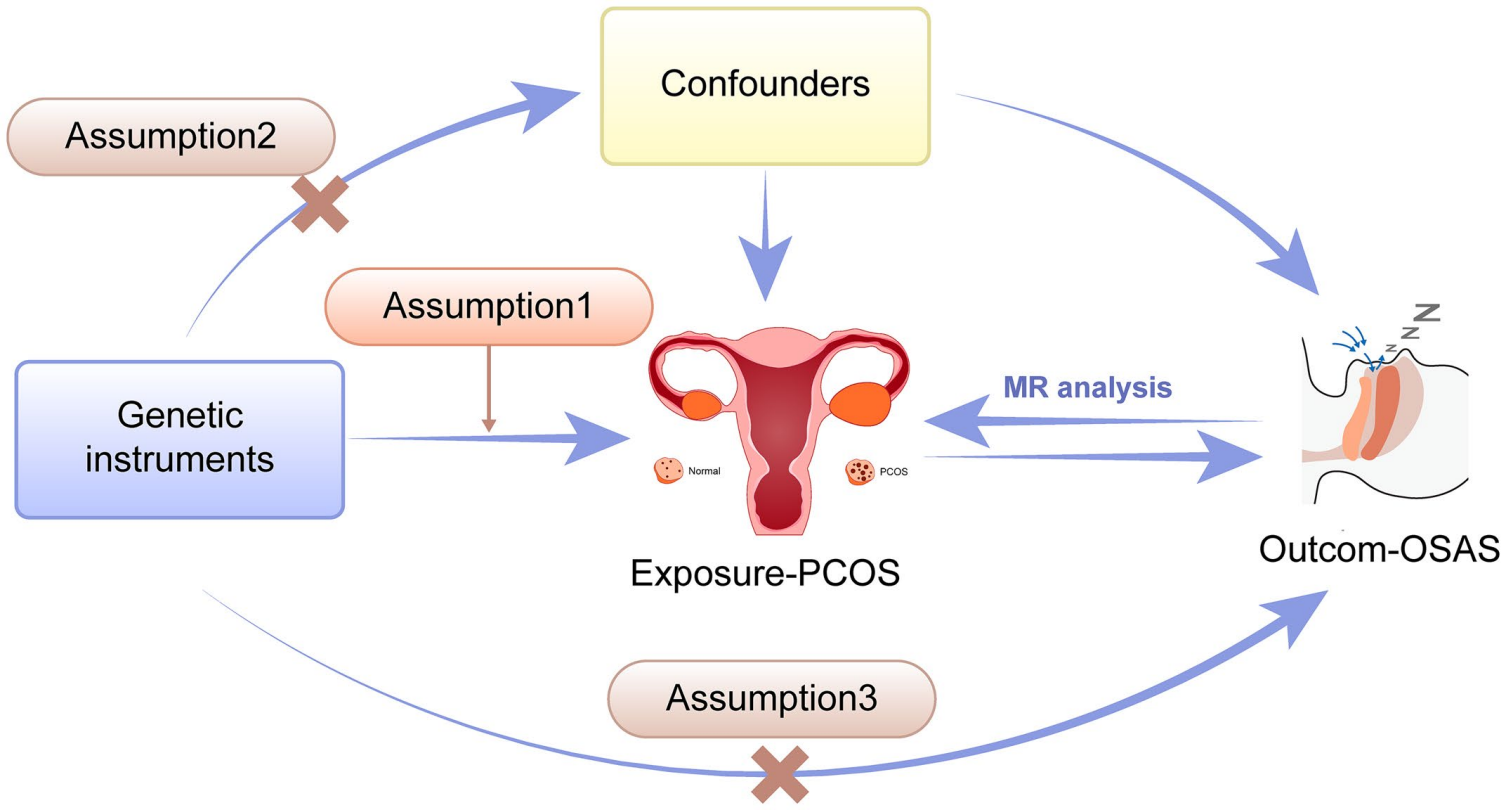
Schematic illustration showing the two-way Mendelian Randomization method employed to investigate the causal connection between PCOS and OSAS. PCOS, Polycystic ovary syndrome; OSAS, Obstructive sleep apnea syndrome.

The two sample Mendelian randomization method was used to examine the potential causal link between PCOS and OSAS, as depicted in Figure 1. For accurate MR conclusions, adherence to three essential prerequisites is crucial: Assumption 1, the IVs must have a strong association with the exposure; Assumption 2, the genetic instruments should have no associations with potential confounding factors. Assumption 3, the impact of the genetic instruments on the outcome should be solely facilitated by the exposure.

### 2.2 Summary statistics from the GWAS for PCOS

The GWAS catalog provided the pooled data for analyzing PCOS, listed under the identifier ebi-a-GCST90044902 (11). This dataset was compiled by identifying PCOS diagnoses through ICD codes (ICD-8 256.90, ICD-9 256.4, and ICD-10 E28.2) within national health records, with the remainder of the female population serving as the control group. The study population, with European genetic backgrounds, comprised 3,609 cases of PCOS and 229,788 control subjects, with adjustments made for participant age to refine the study’s accuracy.

### 2.3 GWAS summary statistics for OSAS

Genetic markers for the OSAS research were obtained from the FinnGen database (12). The identification of OSAS cases utilized the ICD-10 code G47.3, with the condition’s diagnosis requiring clinical evidence and an AHI of ≥5 events per hour. The dataset includes 38,998 subjects with OSAS and 336,659 controls, all of European ancestry, according to the database information.

### 2.4 SNP selection as IVs

In the segment focusing on OSAS, SNPs were chosen as instrumental variables (IVs) following a rigorous criterion of *p* < 5 × 10^−8^. Conversely, for analyzing PCOS as an exposure, a broader selection strategy was adopted for SNPs, applying the threshold of statistical significance for *p* < 5 × 10^−6^ to include a wider range of IVs. We meticulously excluded SNPs that violated the set linkage disequilibrium criterion (*r*^2^ < 0.001 within a 10,000 kb span) (13). SNPs of the *F*-statistic (Beta^2^/SE^2^) below 10, indicating weak instrumental variables, were also removed from our study (14). Given the known confounding influence of obesity and BMI on both OSAS and PCOS, we diligently utilized the PhenoScanner to omit the instrumental variables linked to BMI and obesity, ensuring a more precise analysis of the results (15). PhenoScanner is a publicly accessible database and toolkit that enables users to query a wide range of public genetic datasets to explore associations between specific genotypes and phenotypes.

The concluding assessment pinpointed 13 SNPs as IVs for OSAS and 11 genetic variants for PCOS, employing a selective process that deliberately excluded certain SNPs to ensure only robust IVs were incorporated into the MR study. In summary, the approach rigorously filtered out palindromic SNPs and those showing genome-wide significant associations with the outcome, ensuring a robust selection of IVs.

### 2.5 Analysis of genetic correlations

We carried out a genetic correlation study to investigate the genetic links between OSAS and PCOS, employing Linkage Disequilibrium Score Regression (LDSC) as a key method to measure the genetic overlap and uncover possible common causal pathways between these conditions. To conduct this analysis, we utilized the LDSC software package (version 2.0.0). This tool is specifically designed for estimating heritability and genetic correlation from GWAS summary statistics, leveraging linkage disequilibrium information (16).

### 2.6 The methods for Mendelian randomization

This study utilized a suite of MR techniques, including IVW, weighted median, weighted mode, simple mode, and MR Egger, to examine the possible causative link connecting PCOS with OSAS (13, 17). The IVW approach was selected as the principal technique due to its enhanced statistical efficiency under the premise that all SNPs function as reliable instrumental factors (18). IVW exploits the genetic correlation between IVs, aggregating the accuracy of each estimate and thus prioritizing more accurate measures for dependable causal analysis. The additional methods provided support to IVW, each predicated on distinct assumptions about horizontal pleiotropy, aiming collectively to furnish thorough and solid MR findings under different scenarios (19).

### 2.7 Mendelian randomization assessment

Our analysis was conducted using the R programming environment (version 4.1.2) with the “TwoSampleMR” package. To examine the heterogeneity among instrumental variables, we applied Cochran’s Q analysis as part of the IVW approach. Acknowledging the significance of pleiotropy, where genetic variants influence multiple traits, our study tackled horizontal pleiotropy and identified outliers using the method for residual sum and outlier analysis in Pleiotropy (MR-PRESSO) (20). For detecting directional pleiotropy among IVs, the MR-Egger intercept test was used, where an intercept differing from zero indicates directional pleiotropy (21). Moreover, we undertook a single exclusion method sensitivity analysis to identify any biases induced by specific SNPs, systematically removing each SNP to observe its effect on the findings (22), ensuring a thorough and unbiased assessment of the Mendelian randomization results.

## 3 Results

### 3.1 Genetic association between PCOS and OSAS

In the result of genetic association, we observed a modest genetic link between PCOS and OSAS, indicated by the genetic correlation of 0.03 and the *p* value of 0.77. This minimal genetic overlap suggested that the IVs employed in MR analysis were likely to be specific, enhancing the analysis by minimizing the influence of confounding factors and thereby making the causal inferences drawn from MR more trustworthy (Table 1).

**Table 1:** Genetic association between PCOS and OSAS derived from LDSC regression.

Trait 1	Trait 2	Rg (Se)	Pval
PCOS	OSAS	0.03 (0.11)	0.77

PCOS, Polycystic ovary syndrome; OSAS, Obstructive sleep apnea syndrome; Rg, Genetic correlation; Se, Standard error; Pval, *p* value.

### 3.2 Causal link between OSAS and PCOS through forward MR analysis

**Figure 2:**
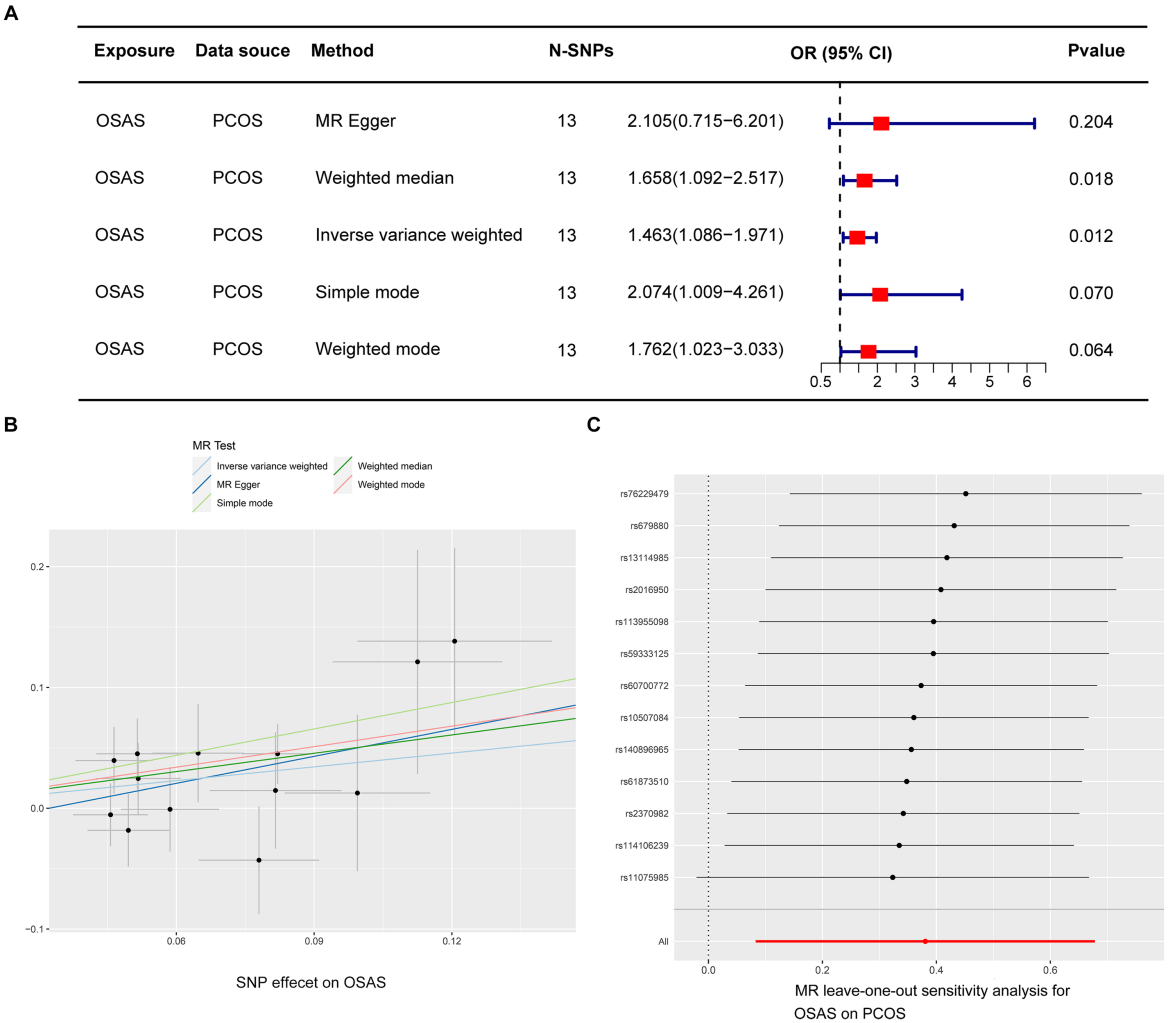
Depicts MR studies on the causal link with OSAS as the predictor and PCOS as the result. **(A)** Shows the causative influence of OSAS on PCOS. **(B)** A scatter diagram showing the analysis of individual SNPs to assess OSAS effect on the likelihood of PCOS. **(C)** A graph excluding one genetic variant shows the combined impact of SNPs on the link connecting OSAS with PCOS.

In the initial stage, we identified SNPs closely linked to OSAS across the genomic significance level, for a particular focus on those exhibiting no linkage disequilibrium. Following a meticulous process that involved the exclusion of SNPs with pleiotropic effects linked to obesity and BMI, we removed specific SNPs (rs10986730, rs11981973, and rs1228509). This rigorous curation ultimately led to the final selection of 13 SNPs, all of which demonstrated an *F*-Statistic surpassing 10, highlighting their robustness as IVs (Supplementary Table S1). In the application of the IVW methodology, our analysis identified an odds ratio (OR) of 1.463, with a 95% confidence interval (CI) extending from 1.086 to 1.971 and a significance level of *p* = 0.012. This indicates a probable causal association between PCOS and OSAS. Furthermore, employing the weighted median approach yielded an OR of 1.658, with a 95% CI ranging from 1.092 to 2.517, and a *p* value of 0.018, reinforcing the potential causal linkage. In contrast, the application of the MR Egger method revealed an OR of 2.105, with a 95% CI from 0.715 to 6.201, and a *p* value of 0.204, while the simple mode approach produced an OR of 2.074, with a 95% CI of 1.009–4.261, and a *p* value of 0.070. Additionally, the weighted mode method indicated an OR of 1.762, with a 95% CI of 1.023 to 3.033, and a *p* value of 0.064, suggesting an ambiguous causal relationship between PCOS and OSAS (Figure 2A). The IVW method is notably advantageous in Mendelian randomization analyses for its capability to efficiently handle multiple SNPs as instrumental variables. This approach preserves the analytical rigor, even in the presence of minor correlations among SNPs. By calculating the average of the causal effect estimates from each SNP through inverse variance weighting, the IVW method significantly enhances the accuracy and reliability of causal determination (23, 24). Our analysis did not uncover significant heterogeneity, as indicated by the Cochran’s *Q*-test, which presented a value of 9.823 with a *p* value of 0.631. Moreover, the MR Egger intercept test did not demonstrate any evidence of directional pleiotropy, with an intercept of −0.024 and a *p* value of 0.506 (Table 2). The findings were graphically depicted in scatterplots in Figure 2B. Additionally, the leave one out method scrutiny confirmed that none specific SNP disproportionately affected the estimated consequence of PCOS on OSAS risk (Figure 2C).

**Table 2:** PCOS and OSAS: heterogeneity and pleiotropy analysis.

Exposure	Outcome	Heterogeneity		MR-egger intercept	
		Cochrane’s Q	Heterogeneity (pval)	Egger-intercept	Pleiotropy (pval)
OSAS	PCOS	9.823	0.631	−0.024	0.506
PCOS	OSAS	7.756	0.653	0.001	0.805

### 3.3 Causal connection between PCOS and OSAS through reverse MR analysis

In our reverse MR study, we investigated the potential of PCOS to cause OSAS, specifically by removing SNPs linked to obesity and increased BMI. After a thorough process that meticulously screened for SNPs with pleiotropic effects associated with obesity and BMI, two specific SNPs, namely rs804263 and rs71562896, were excluded. As a result of this refinement, IVs were narrowed down to a total of 11 specific SNPs (Supplementary Table S2), Utilizing the IVW methodology, our analysis substantiated a significant causal relationship between PCOS and OSAS, evidenced by an OR of 1.041 within a 95% CI ranging between 1.012 and 1.072, achieving a *p* value of 0.006. The weighted mode analysis indicated an OR of 1.028 with a 95% CI between 0.982 and 1.076, resulting in a *p* value of 0.266; the MR Egger technique reported an OR of 1.036 with a 95% CI between 0.987 and 1.087, with a *p* value of 0.186; the simple mode method exhibited an OR of 1.028 with a 95% CI between 0.971 and 1.088, and a *p* value of 0.366; and the weighted median approach showed an OR of 1.029 with a 95% CI between 0.986 and 1.073, with a *p* value of 0.187.

**Figure 3:**
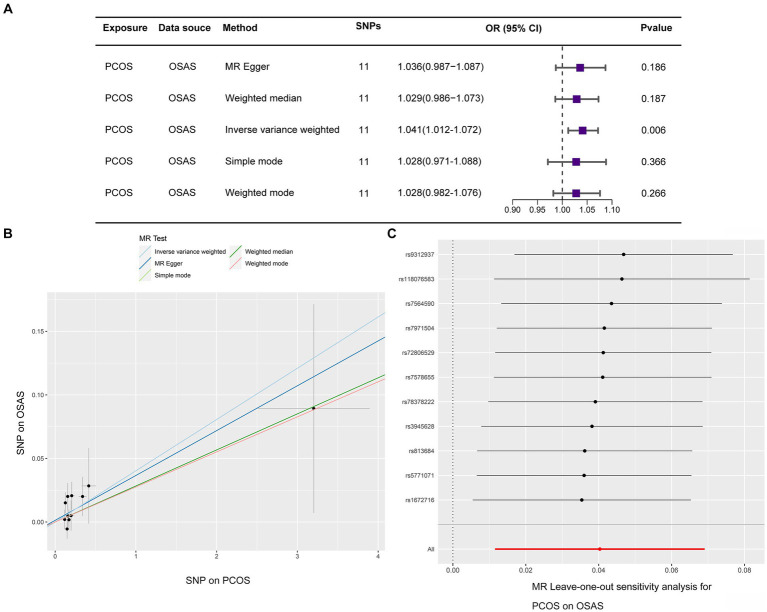
MR evaluations reveal causal relationships, with PCOS predicting OSAS the consequence. **(A)** Details the causative effect of PCOS on OSAS. **(B)** A scatter diagram examines the SNPs to understand how PCOS affects the onset OSAS. **(C)** A leave one out graph underscores the collective effects of SNPs and their impact link connecting PCOS and OSAS.

The primary analysis using the IVW method, depicted in Figure 3A, established a causal link between PCOS and OSAS. Cochran’s *Q*-test indicated homogeneity across studies with a *Q*-value of 7.756 and a *p* value of 0.653, suggesting no substantial differences. The MR-Egger intercept analysis presented an intercept of 0.001 with a *p* value of 0.805, indicating no evidence of directional pleiotropy, which implies minimal influence of pleiotropic effects on the results. The findings were graphically depicted in scatterplots in Figure 3B. Additionally, the leave one out method scrutiny confirmed that none specific SNP disproportionately affected the estimated consequence of PCOS on OSAS risk (Figure 3C).

## 4 Discussion

This is the inaugural investigation to deeply analyze the causative association between PCOS and OSAS by means of the utilization of the two-sample Mendelian randomization approach. Concentrating on European ancestry subjects, it unveils the intricate dynamics between the two conditions. It provides crucial revelations about the interaction of PCOS and OSAS, underscoring their significant connection. As healthcare challenges continue to evolve, our research brings to light a new understanding of how OSAS and PCOS are interconnected, offering key insights that could lead to improved diagnostic accuracy and treatment options.

Our results affirm that PCOS notably boosts the risk of OSAS, establishing a direct connection that persists even after adjusting for obesity and BMI, consistent with prior research findings (25, 26). Obesity poses a substantial risk factor in the relationship between PCOS and OSAS, with controversy still existing over whether non-obese PCOS leads to OSAS. Obesity is also a frequent coexisting condition among women suffering from PCOS, not only increasing the metabolic burden on the body but also serving as one of the main mechanisms of the syndrome’s pathogenesis (27). The accumulation of fat tissue around the upper airway increases its collapsibility, while upper-body obesity can decrease lung volume and negatively impact respiratory regulation (28). Studies reveal a link between PCOS and an increased likelihood of OSAS, especially pronounced among obese women. Nevertheless, this risk significantly diminishes in non-obese women with PCOS. The research found a 44.4% prevalence of OSAS in obese women with PCOS vs. a 5.5% prevalence in an obese control group with similar BMI levels (*p* = 0.008), indicating that obesity plays a crucial part in the interplay between PCOS and OSAS (29). However, other randomized controlled trials (RCTs) have suggested that patients with PCOS who are not overweight also have a high probability of developing OSAS. Tasali et al. documented a frequency of OSAS among women with PCOS at 56%, considerably greater than the 19% in the non-PCOS control group, a risk that remained even after controlling for BMI, age, and ethnicity (30). A substantial population-based cohort investigation from the United Kingdom demonstrated that the likelihood of developing OSAS was higher in women with PCOS regardless of being of normal weight, overweight, or obese, compared to age, BMI, and geographically matched non-PCOS control women (31). This is consistent with our Mendelian randomization analysis results, indicating a correlation between PCOS and OSAS, even in non-obese women, with obesity exacerbating this correlation.

Our findings indicate that OSAS may play a role in the onset of PCOS, emerging as a potential risk factor for PCOS beyond the effects of obesity and elevated BMI. Research has established a connection between PCOS and a state of mild chronic inflammation, where a complex interplay of hyperinsulinemia, hyperandrogenism, and inflammation perpetuates the disorder’s pathophysiology in a vicious cycle (32). In individuals with OSAS, the recurrent episodes of apnea, coupled with cycles of hypoxia and reoxygenation, lead to the release of substantial inflammatory mediators (33). These inflammatory and oxidative stress responses further exacerbate the pathophysiological mechanisms underlying PCOS. Notably, OSAS enhances both muscle and cardiac sympathetic nerve activity, which are pivotal in the development of PCOS by fostering chronic inflammation, inducing changes in ovarian function, and facilitating the polycystic transformation of ovarian morphology (34). Studies involving rodent models for polycystic ovary syndrome demonstrated that an increase in sympathetic ovarian nerve activity and a rise in nerve growth factor synthesis within the ovaries contribute to the pathology’s onset and progression (35). Further research has confirmed that patients with PCOS show greater cardiac rhythm variability, diminished heart rate recuperation following exercise, and heightened muscle and cardiac sympathetic nerve activity, even when controlling for age and BMI (29). Ibrahim et al. have indicated that individuals with OSAS face a higher risk of infertility and miscarriage (36). This elevated risk may stem from the association of OSAS with an increased likelihood of developing polycystic ovary syndrome (PCOS), which is known to contribute to female infertility. This corresponds with the conclusions drawn from our Mendelian randomization analysis, which indicate that OSAS could participate in the development of PCOS, impacting women regardless of their obesity status, with obesity amplifying this relationship.

High androgen levels in PCOS may lead to alterations in the upper airway anatomy, predisposing patients to OSAS. Conversely, OSAS may directly or via the link to insulin resistance and decreased sex hormone-binding protein levels, play a part in increasing androgen levels in PCOS (37). Moreover, the increased sympathetic nerve activity and oxidative stress associated with PCOS can promote insulin resistance, with OSAS exacerbating these metabolic abnormalities through similar mechanisms (38). Thus, a vicious cycle between PCOS and OSA emerges, posing significant health risks. Firstly, it compromises sleep quality; sleep quality is impacted in both OSA and PCOS patients, with those suffering from both conditions experiencing more severe effects (39). Secondly, it may lead to sexual dysfunction and infertility: both OSAS and PCOS affect sexual function, with PCOS women reporting lower levels of sexual satisfaction compared to those without PCOS (40). The inflammation, oxidative stress, and increased sympathetic excitability caused by OSAS, along with fragmented sleep and abnormal sleep architecture, can reduce the secretion of gonadotropins and gonadotropin-releasing hormones, thus impairing reproductive function and leading to infertility in PCOS patients. Thirdly, it amplifies the risk of metabolic disorders: studies indicate that women suffering from both PCOS and OSAS experience more pronounced metabolic imbalances, such as increased fasting blood sugar, diminished glucose tolerance, and reduced insulin sensitivity, thereby increasing their susceptibility to metabolic diseases (41). Finally, it escalates the risk of cardiovascular incidents: women with both PCOS and OSAS display increased levels of cardiac contraction and relaxation pressure levels, along with triglyceride concentrations relative to individuals not afflicted with OSAS, a disparity that persists even when controlling for BMI (42).

Hence, future research should not only explore the actual incidence of OSAS among a more indicative sample of females diagnosed with PCOS and compare incidences across different ethnic groups but also ensure that women with OSAS undergo pelvic ultrasound examinations for early treatment. It is advisable to screen women with PCOS for sleep-related breathing disorders and provide them with appropriate treatment as needed. Continuous positive airway pressure can alleviate symptoms in individuals with OSAS, and improve insulin function, oxidative stress, and sympathetic excitability, thereby reducing blood pressure and decreasing catecholamine levels (43).

However, it is important to acknowledge the restrictions present in our investigation. Although our bidirectional MR analysis sheds light on the PCOS-OSAS causal relationship, the evidence from the various MR methods used does not consistently support a direct causal link between these conditions. Future research could be enriched by implementing clinical RCTs to delve deeper into and confirm the dynamics of the relationship between PCOS and OSAS. Furthermore, our reliance on publicly available summary statistics limited our ability to include comprehensive demographic variables such as age, gender, and comorbidities in our analysis, thus representing a notable limitation in our approach. Nevertheless, the strength of MR analysis lies in its ability to apply genetic instruments to elucidate possible causative links between exposures and outcomes, eliminating the necessity for intricate demographic details. Despite these acknowledged limitations, our study represents a substantial stride in enhancing comprehension of the genetic associations and causal connections between PCOS and OSAS. This work lays a strong basis for future pursuits in diagnostics and preventive strategies targeting these disorders.

## 5 Conclusion

Our study conclusively establishes the bidirectional causal link between PCOS and OSAS, underlining the complexity of their interaction. Through Mendelian Randomization analysis, we demonstrated significant causal links from OSAS to PCOS and vice versa, with robust odds ratios confirming these associations. Our study paves the way for developing targeted screening and preventative strategies, emphasizing the need to address both conditions concurrently in clinical practice.

## Data availability statement

The original contributions presented in the study are included in the article/Supplementary material; further inquiries can be directed to the corresponding authors.

## Ethics statement

Our investigations utilized data from published research or GWAS summaries that are in the public domain. These studies received clearance from relevant ethics committees, negating the need for further ethical consent.

## Author contributions

PL: Conceptualization, Investigation, Methodology, Writing – original draft. QZ: Formal analysis, Investigation, Writing – original draft. HD: Validation, Visualization, Writing – review & editing. HZ: Funding acquisition, Software, Writing – review & editing.
