# Supplementary material for: Association of obstructive sleep apnea syndrome with polycystic ovary syndrome through bidirectional Mendelian randomization
Source: Front Med (Lausanne). 2024 Jun 28;11:1429783. doi: 10.3389/fmed.2024.1429783 (PMC11239387; doi:10.3389/fmed.2024.1429783)
Supplement: Supplementary file 1 [file Table_1.doc]

**TableS1 | Comprehensive details of the SNPs utilized in the MR analysis of OSAS on PCOS**

| SNP | effect_allele | other_allele | beta.exposure | beta.outcome | eaf.exposure | se.exposure | pval.exposure |
| --- | --- | --- | --- | --- | --- | --- | --- |
| rs10507084 | T | C | 0.0647465 | 0.0456 | 0.179407 | 0.00996658 | 8.23E-11 |
| rs11075985 | A | C | 0.0820534 | 0.0451 | 0.428739 | 0.00771867 | 2.15E-26 |
| rs113955098 | A | G | -0.099427 | -0.0126 | 0.0674969 | 0.0158313 | 3.38E-10 |
| rs114106239 | T | C | -0.120592 | -0.1383 | 0.0371118 | 0.0211507 | 1.19E-08 |
| rs13114985 | G | T | 0.0456779 | -0.0054 | 0.33828 | 0.00814065 | 2.01E-08 |
| rs140896965 | T | C | -0.112519 | -0.1212 | 0.0490272 | 0.0184655 | 1.11E-09 |
| rs2016950 | T | C | -0.0586327 | 9.00E-04 | 0.157983 | 0.0106896 | 4.13E-08 |
| rs2370982 | T | C | 0.0515131 | 0.0451 | 0.238198 | 0.00892902 | 7.97E-09 |
| rs59333125 | C | A | -0.0815898 | -0.0147 | 0.0807381 | 0.0143562 | 1.32E-08 |
| rs60700772 | C | T | 0.0516943 | 0.0246 | 0.221225 | 0.00923826 | 2.20E-08 |
| rs61873510 | T | G | 0.0464319 | 0.0395 | 0.301035 | 0.0084028 | 3.28E-08 |
| rs679880 | A | G | 0.0495476 | -0.0183 | 0.745023 | 0.00889332 | 2.53E-08 |
| rs76229479 | C | A | -0.0779901 | 0.043 | 0.0987789 | 0.0131014 | 2.64E-09 |
