# Supplementary material for: Association of obstructive sleep apnea syndrome with polycystic ovary syndrome through bidirectional Mendelian randomization
Source: Front Med (Lausanne). 2024 Jun 28;11:1429783. doi: 10.3389/fmed.2024.1429783 (PMC11239387; doi:10.3389/fmed.2024.1429783)
Supplement: Supplementary file 2 [file Table_2.doc]

**TableS2 | Comprehensive details of the SNPs utilized in the MR analysis of PCOS on OSAS**

| SNP | effect_allele | other_allele | beta.exposure | beta.outcome | eaf.exposure | se.exposure | pval.exposure |
| --- | --- | --- | --- | --- | --- | --- | --- |
| rs118076583 | T | C | 3.2024 | 0.0892581 | 0.00227451 | 0.6917 | 3.66E-06 |
| rs1672716 | G | A | -0.2057 | -0.0206817 | 0.145232 | 0.0359 | 9.81E-09 |
| rs3945628 | C | T | 0.3397 | 0.0200656 | 0.0656637 | 0.0487 | 2.95E-12 |
| rs5771071 | T | G | 0.1556 | 0.0200845 | 0.155498 | 0.0336 | 3.56E-06 |
| rs72806529 | A | G | -0.1977 | -0.0050242 | 0.118228 | 0.0392 | 4.58E-07 |
| rs7564590 | T | C | 0.171 | 0.00176597 | 0.345519 | 0.026 | 4.84E-11 |
| rs7578655 | A | C | 0.1583 | 0.0047755 | 0.267504 | 0.0293 | 6.37E-08 |
| rs78378222 | G | T | 0.4163 | 0.028412 | 0.0172954 | 0.0899 | 3.62E-06 |
| rs7971504 | G | A | 0.1178 | 0.00201022 | 0.549505 | 0.0254 | 3.44E-06 |
| rs813684 | T | C | -0.1268 | -0.0150721 | 0.686978 | 0.0276 | 4.23E-06 |
| rs9312937 | C | T | 0.1482 | -0.00548444 | 0.419608 | 0.0263 | 1.79E-08 |
